# Editorial: AI and neuroscience: integrating knowledge, reasoning, and theory of mind

**DOI:** 10.3389/fncom.2026.1859797

**Published:** 2026-05-19

**Authors:** Darren J. Edwards, Bochao Zou, Rob Lowe, Andrew Owens

**Affiliations:** 1 Department of Public Health, Swansea University, Swansea, United Kingdom; 2 School of Computer and Communication Engineering, University of Science and Technology, Beijing, China; 3 School of Psychology, Swansea University, Swansea, United Kingdom; 4 Jaguar Land Rover, Warwick, United Kingdom

**Keywords:** artificial intelligence, cognition, computation, neuroscience, reasoning

Artificial intelligence and neuroscience have been converging on a shared problem about how to explain and ultimately build systems (biological or synthetic) that can acquire knowledge from experience, reason under uncertainty, and coordinate perspectives from self-model representations to other minds (Hassabis et al., 2017; Langley et al., 2022; Limanowski and Blankenburg, 2013; Nawaz et al., 2025). The “*AI and neuroscience: integrating knowledge, reasoning, and theory of mind*” theme captures this convergence by explicitly highlighting a broad body of research linking accounts of neural information processing with computational architectures that can learn, generalize, and remain interpretable. At a broad level, the contributions in this Research Topic can be read as collectively operating across three complementary levels. First, they investigate biological substrates of computation and the representational constraints that come with real neural tissue. Second, they advance architectures and modeling frameworks that treat cognition as an evolving repertoire of learned competencies rather than a set of isolated tasks. Third, they address human–AI coupling, i.e., how AI systems can extend cognition without displacing the very internal knowledge structures that make reasoning and perspective-taking possible in the first place.

## A summary of the article contributions

Edwards extends the functional contextual *N*-Frame model (Edwards, 2023, 2024) as an integrative theory linking predictive coding (Friston, 2018; Spratling, 2017), QBism-style observer dependence (Fuchs et al., 2014), and evolutionary dynamics to explain how belief updating and decision-making in humans (and potentially AI) can be modeled within a quantum-cognitive functional contextual formalism. The paper positions consciousness as an active, context-sensitive participant in “actualization” (bridging quantum potentiality to classical outcomes), uses cognitive fallacies (e.g., conjunction effects) as structured signatures of quantum context-dependent inference rather than mere cognitive classical irrationality, and proposes testable boundaries/parameters relevant to AI consciousness beyond standard AI benchmark performance. In the Research Topic's terms, it offers a unifying scaffold for knowledge (context-shaped representations), reasoning (state updates under constraints), and the foundations needed for theory of mind (self-referential observer modeling), while explicitly connecting these claims to experimentally oriented predictions and formal operator-style descriptions, in order to potentially develop safe and ethical AI.

Tütüncü and Gonzalez-Franco argues that “algorithmic suffering” is best treated as a comparative neuroscientific lens rather than as a claim that machines literally feel pain. They identify parallels between human and AI cognition in reward prediction error, Bayesian belief updating, and risk anticipation, while emphasizing a decisive asymmetry: in humans, prediction errors can become globally integrated into a conscious self-model and experienced as threats to meaning and integrity, whereas in current AI they remain numerical updates without phenomenology. Framed within this Research Topic, the paper helps clarify where AI's performance (its observable behavior and outputs) may mimic cognition while still falling short of the selfhood, meaning, and experiential integration that would be required for genuinely human mind-like understanding.

Küchler et al. provide a clear “wetware-to-logic” bridge by demonstrating that engineered *in vitro* neuronal networks with controlled topology can implement basic Boolean operations (including NAND/OR) using stimulation and readout on high-density microelectrode arrays. Importantly, the work goes beyond a proof-of-principle gate: it interrogates encoding and decoding choices (rate-based vs. spike-timing-based schemes such as TTFS), highlighting how representational format constrains how information can be reliably encoded, transmitted, and thus computed. Such minimal networks may serve as building blocks for hybrid intelligence and biocomputing. In the context of this Research Topic, the paper grounds “knowledge and reasoning” in the physical realities of neural signaling and frames neural computation as an input–output mapping that can be characterized and engineered.

Chowdhury et al. review the rapidly developing landscape of inner speech recognition (ISR), i.e., the decoding of covert/inner speech from neural signals, and position machine learning as the primary driver of progress across the ISR pipeline (signal acquisition, preprocessing, feature extraction, and classification). The review synthesizes cognitive models of inner speech alongside a comparative analysis of machine learning (ML) and deep learning (DL) approaches [e.g., support vector machines (SVMs) and random forests vs. convolutional neural network (CNN-based models)], while emphasizing persistent barriers such as signal-to-noise limitations, inter-subject variability, interpretability, and challenges for real-time deployment. Conceptually, inner speech sits at the intersection of language, self-regulation, and planning; as such, ISR research provides a natural bridge toward higher-order cognitive functions (i.e., neural signals → inner speech decoding → internal cognition). In the context of this Research Topic, the paper can be read as exemplifying the “integrating knowledge and reasoning” theme as an applied neuroscience-to-AI translation problem with significant clinical and assistive-technology implications.

Prudkov advances a hypothesis-and-theory account of AGI grounded in the “goals–means correspondence,” arguing that the central challenge for general intelligence is not merely producing competent outputs, but establishing and dynamically maintaining the correspondence between goals and the means available in an evolving world. The paper critiques two conventional agent architectures in the form of those with jointly specified goals and means at “birth” (that is, at the moment the agent is created or initialized), and those in which goals and means are constructed separately. It proposes an alternative: the joint construction of arbitrary goals and means under a criterion of minimal construction cost, framed as a cognitive analog of least action. This proposal is explicitly developmental (agents “grow” by altering structure), and it connects naturally to neuroscience-informed views of planning and prefrontal control, while supplying a formal lens on how reasoning can emerge as a type of goal-directed process. Within this Research Topic, it can be read as contributing a unifying architectural principle for linking learning, reasoning, and adaptive agency.

O'Sullivan et al. introduce “Affinity,” a visual analytics tool designed to make computational models of stimulus equivalence and derived relational responding more transparent and experimentally useful. Built on Enhanced Equivalence Projective Simulation, the tool provides real-time visualizations of the evolving relational memory of an agent and operationalizes Relational Density Theory (Belisle and Dixon, 2020) by modeling higher-order network properties (e.g., density, volume, and mass) that may explain resistance to change and the dynamics of relational learning. As a contribution to “knowledge and reasoning,” this work is significant because relational generalization and abstraction are foundational to language-like cognition. More broadly, the framework connects computational modeling of relational learning with cognitive and behavioral science accounts of flexible, context-sensitive responding. Affinity's (their visual analytics tool) emphasis on interpretability and experiment-as-interface also aligns with a wider shift toward explainable AI, where models are designed not only to perform but to reveal their internal process dynamics in ways that can be systematically analyzed and compared to empirical data.

Klein and Klein broaden the scope from building intelligent systems to preserving and redesigning environments to support human cognition in AI-rich contexts. Their “extended hollowed mind” framework argues that generative AI produces a dual outcome in education: it can extend cognition while also enabling a “cognitive bypass” that weakens the internal structures required for deep learning and evaluative judgment, leading to a lack of deep cognitive understanding. They introduce the term “Sovereignty Trap” (whereby humans have the tendency to cede judgment to an authoritative system and trust AI too much) and reframe the educational target as a “Fortified Mind,” defined as an internal architecture of knowledge and metacognitive skills necessary for cognitive sovereignty (whereby humans develop solid knowledge and critical thinking skills). The paper's relevance to this Research Topic is 2-fold: it anchors the discussion in cognitive science and neurobiological constraints on effortful reasoning, and it treats reasoning capacity as an internal architecture that must be actively trained. It therefore functions as a crucial boundary condition on the design of AI systems for learning, i.e., tools that replace human reasoning risk degrading the very cognitive competencies required to interpret, validate, and effectively use them.

Finally, Johansson and Hammer review the Machine-Psychology program implemented in the Non-Axiomatic Reasoning System (NARS), presenting a staged developmental roadmap from operant learning to abstraction, functional equivalence, and arbitrarily applicable relational responding. A central strength of this work is its explicit mapping between architectural mechanisms (e.g., temporal inference, resource limitations, relational generalization) and well-established psychological phenomena, treating cognition as a learnable process rather than as a collection of task-specific solutions. The manuscript also links relational learning and contextual control to perspective-taking and theory-of-mind–related abilities, situating cognitive architecture as a bridge between neuroscience-relevant process models and AGI ambitions. In the context of this Research Topic, it directly embodies the integrative thesis: knowledge emerges through experience, reasoning operates under bounded resources, and higher social-cognitive competencies can be built from tractable learning primitives.

In conclusion, taken together, these contributions sketch a coherent multi-scale picture of integration. At the substrate level, engineered neuronal networks clarify what computation in biological substrates can look like, and why representational choices (e.g., rate vs. timing, i.e., how often neurons fire, such as 50 spikes per second vs. when they fire, such as precise millisecond patterns, order, synchrony) matter for reliable inference. At the systems level, architectural proposals and interpretable modeling tools advance a view of intelligence as developmental: learned competencies accumulate from simple feedback-driven adaptation to symbolic and relational generalization. At the human interface level, the educational and epistemic framing reminds us that theory-of-mind-related, reasoning-enabled AI will only be beneficial if humans retain the internal knowledge structures required to evaluate, contextualize, and govern it.

From this Research Topic, a shared message emerges. Societal progress and wellbeing in the age of AI clearly come less from chasing isolated benchmarks and more from building process bridges: between neural codes and computation, between learning histories and relational abstraction, and between tool design and the preservation of human cognitive sovereignty. This is precisely the integration challenge at the heart of “*AI and neuroscience: integrating knowledge, reasoning, and theory of mind*” and we hope it will lead to a new generation of intelligent systems designed not only for performance but for the advancement of a fairer, healthier, and more resilient society.
